# HIV-1 Infection Transcriptomics: Meta-Analysis of CD4+ T Cells Gene Expression Profiles

**DOI:** 10.3390/v13020244

**Published:** 2021-02-04

**Authors:** Antonio Victor Campos Coelho, Rossella Gratton, João Paulo Britto de Melo, José Leandro Andrade-Santos, Rafael Lima Guimarães, Sergio Crovella, Paola Maura Tricarico, Lucas André Cavalcanti Brandão

**Affiliations:** 1 Department of Pathology, Federal University of Pernambuco (UFPE), Av. Prof. Moraes Rego, 1235 Cidade Universitária, Recife 50670-901, Brazil; joaobritto26@gmail.com (J.P.B.d.M.); lucabrand@gmail.com (L.A.C.B.); 2 Department of Advanced Translational Microbiology, Institute for Maternal and Child Health IRCCS Burlo Garofolo, Via dell’Istria 65/1, 34137 Trieste, Italy; rossella.gratton@gmail.com (R.G.); tricaricopa@gmail.com (P.M.T.); 3 Department of Genetics-Federal, University of Pernambuco (UFPE), Av. Prof. Moraes Rego, 1235 Cidade Universitária, Recife 50670-901, Brazil; jlandrades19@gmail.com (J.L.A.-S.); rafaellg@gmail.com (R.L.G.); 4 Laboratory of Immunopathology Keizo Asami (LIKA), Federal University of Pernambuco (UFPE), Av. Prof. Moraes Rego, 1235 Cidade Universitária, Recife 50670-901, Brazil; 5 Department of Biological and Environmental Sciences, College of Arts and Sciences, University of Qatar, Doha P.O. Box 2713, Qatar; sgrovella@qu.edu.qa

**Keywords:** infection, latency, transcriptomics, genomics, gene ontology, pathway analysis

## Abstract

HIV-1 infection elicits a complex dynamic of the expression various host genes. High throughput sequencing added an expressive amount of information regarding HIV-1 infections and pathogenesis. RNA sequencing (RNA-Seq) is currently the tool of choice to investigate gene expression in a several range of experimental setting. This study aims at performing a meta-analysis of RNA-Seq expression profiles in samples of HIV-1 infected CD4+ T cells compared to uninfected cells to assess consistently differentially expressed genes in the context of HIV-1 infection. We selected two studies (22 samples: 15 experimentally infected and 7 mock-infected). We found 208 differentially expressed genes in infected cells when compared to uninfected/mock-infected cells. This result had moderate overlap when compared to previous studies of HIV-1 infection transcriptomics, but we identified 64 genes already known to interact with HIV-1 according to the HIV-1 Human Interaction Database. A gene ontology (GO) analysis revealed enrichment of several pathways involved in immune response, cell adhesion, cell migration, inflammation, apoptosis, Wnt, Notch and ERK/MAPK signaling.

## 1. Introduction

The human immunodeficiency virus type 1 (HIV-1) is the etiological agent of acquired immunodeficiency syndrome (AIDS). HIV-1 infection is still prevalent, with 1.7 million new cases and 690,000 deaths in 2019 [1]. HIV-1 infection is known to elicit a complex dynamic of various host genes expression [2].

High throughput sequencing contributed to a meaningful amount of information regarding HIV-1 infections and pathogenesis. RNA sequencing (RNA-Seq) is currently the tool of choice to assess gene expression in a range of experiments with distinct conditions or cell types [3].

HIV-1 infection is a complex multifactorial phenomenon, making it suitable to transcriptomic analyses for the understanding of the viral pathogenesis, allowing the detection of altered cellular pathways during all phases of the infectious process [2].

In this article we performed a meta-analysis of RNA-Seq expression profiles in samples of HIV-1 CD4+ T infected cells compared to uninfected cells to assess consistently differentially expressed genes in the context of HIV-1 infection.

## 2. Materials and Methods

### 2.1. Study Search Strategy

We searched the Sequence Read Archive (SRA) [4] for studies involving RNA-Seq in the context of HIV-1 infection through Entrez Direct, National Center for Biotechnology Information (NCBI)’s command line utility [5]. We looked for titles and abstracts containing the keywords “HIV”, “HIV-1”, “HIV-infection”, “HIV positive” and variations thereof in the Bioprojects database division of the SRA. The results were filtered to include only studies in human samples (*Homo sapiens* organism filter) and transcriptome gene expression (RNA-Seq). We did not consider gene expression microarray (“chip”) studies in order to work with comparable findings.

The results of the search were curated to identify HIV-1 infection experiments involving primary CD4+ T cells and their associated publications via PubMed id (PMID). The publications were downloaded for further review. The curation process was performed by, at least, two independent readers prior to inclusion.

### 2.2. RNA-Seq Data Collection, Processing and Meta-Analysis

The raw sequencing reads (.fastq) files were downloaded through Entrez Direct [5]. Only the sequencing reads needed for the meta-analysis objectives were downloaded. For time-repeated studies, we downloaded the runs for the last time point only.

The reads were re-processed using Trimmomatic software v0.39 [6] to trim Illumina adapters and to exclude reads counting fewer than 25 bases. Then, the remaining reads were mapped on the National Center for Biotechnology (NCBI) human GRCh38 reference genome and sorted by coordinates using STAR aligner [7]. Aligned reads (BAM files) were imported into R software and processed with the *Rsubread* package [8], whose *featureCounts* function mapped sequencing reads to genomic features using an in-built human GRCh38 genome annotation (28,395 genes), quantifying raw expression levels per gene per sample, producing a gene count table for each sample. All subsequent analyses were made with R software version 4.0.2 [9].

The gene count tables were then converted into a *DESeq2* package [10] object. During this process, the counts from technical replicates were collapsed into single count per unique sample.

The *RankProd* package for R software was used to perform the meta-analysis. The package performs the rank product (RP) and rank sum tests, non-parametric tests that detect consistently differentially expressed genes in independent and replicated experiments. We adopted the meta-analysis methodology derived from a previously published study [11].

Then, we filtered the meta-analysis results to obtain a list of meta-analysis differentially expressed genes (maDEGs) meeting the following criteria: (1) pooled, percentage of false prediction (pfp)-adjusted *p*-value < 0.05 and (2) |log2(fold-changes)| > 1.

Gene names (symbols) were derived from gene ids with *annotate* [12] and *org.Hs.eg.db* [13] packages.

### 2.3. Gene Ontology Enrichment Analysis

Following the identification of DEGs via meta-analysis as described above, we performed a gene ontology (GO) enrichment analysis through the *goana* function of the *limma* package [14]. The GO ids which (1) belong to biological processes (BP) ontology; (2) have FDR-adjusted enrichment test *p*-value < 0.05; and (3) are related to at least five DEGs identified in the step above were considered to be enriched pathways during HIV-1 infection.

### 2.4. Cross-Referencing and Set Analysis of DEGs

Following the production of the list of DEGs by the meta-analysis, we compared it to previous studies involving HIV-1 replication and life-cycle [15,16,17,18] and data deposited in public databases, such as the HIV-1 Human Interaction Database [19,20,21] and RNAcentral, a database of non-coding RNA [22].

We created a cross-reference table using structured query language (SQL) to extract genes in common from all sources. Therefore, we could identify consistently associated genes as well as new candidates involved in HIV-1 infection.

To assess if the observed intersection of the meta-analysis list with the other sources is within the expected by random chance, we performed simulations to determine an expected intersection number by producing empirical simulations using “mock genes”, a list of random but unique strings to represent gene symbols with the ids package of R software [23] (Script S1).

First, we produced a list of 28,395 unique random strings to represent our annotated human genome. From this list, we randomly sampled two independent sets. The first one contained *D* elements, representing our DEG list. The second one contained *O* elements, where *O* was the size of the original genes list reported by each source. The process of formation of these two sets was repeated 10,000 times. Each time, the two sets were different from the previous ones, and the number of overlapping genes (intersection) was calculated.

Thus, we obtained 10,000 intersection values. The median of these values was considered the expected number. Therefore, the binomial distribution was used to test the observed intersection number with this expected number via one-sided tests under the null hypothesis that the intersection of the original source with the meta-analysis list is equal or less than expected by chance. If *p*-value < 0.05, we would reject the null hypothesis and assume that our DEG list had a higher concordance with the source than expected by chance.

## 3. Results

The search strategy resulted in 94 experiment abstracts. Among those, nine had experiments involving primary CD4+ T cells. Four among these nine involved experiments regarding HIV-1 infection. Two studies with a total of 22 unique samples were included in the meta-analysis. The remaining seven were removed due to absence of publication, preventing assessment of the study design (*n* = 1), the authors sequenced just non-coding RNA, preventing comparability (*n* = 1), absence of biological replicates in the sample (*n* = 1), or because they involved testing of drugs for HIV-1 latency reactivation (*n* = 4).

The first study, from Langer et al. (PRJNA482835, GSE117655) [24] infected CD4+ T cells collected from four independent HIV-1 negative donors with three different HIV-1 primary isolates in vitro. The RNA of infected cells was collected 72 h post-infection and sequenced.

The second study, by Shytaj et al. (PRJNA524856, GSE127468) [25], collected primary CD4+ T cells from total blood of three HIV-1 negative donors (referred to as donor 14, donor 49 and donor 50). After isolation, CD4+ T-cells were activated for 72 h. Following activation, cells were divided in two groups and either infected with HIV-1 NL4-3 isolate or mock-infected. Total RNA extraction was performed at 3, 7, 9 and 14 days post-infection for subsequent RNA-Seq. The 3 days post-infection samples were selected for further analysis.

Overall, 22 unique samples were selected for inclusion in the meta-analysis, 15 being experimentally infected and 7 mock-infected (Table 1 and Table S1).

**Figure 1:**
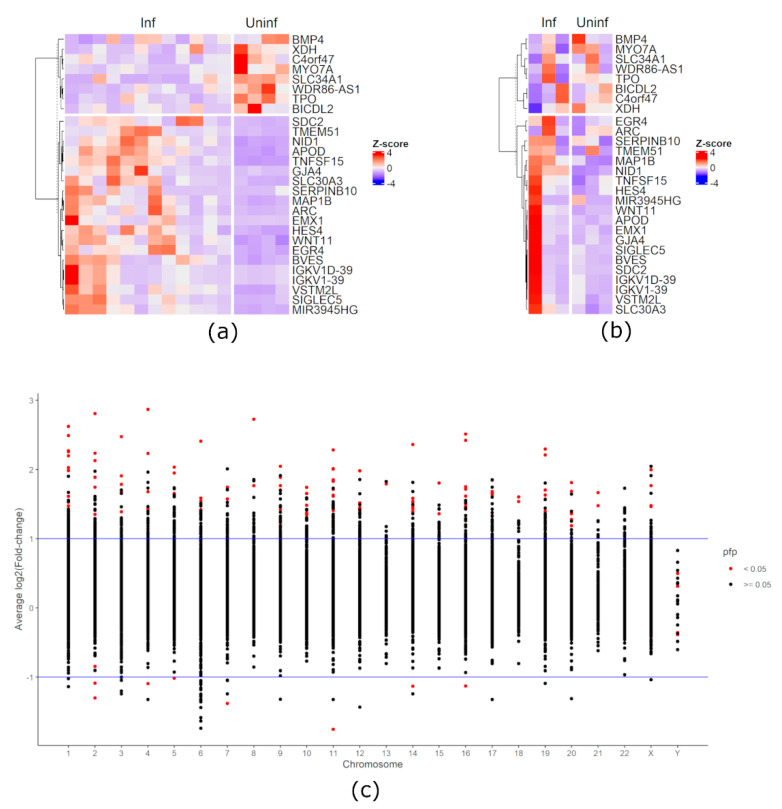
Trimmed mean of M-values (TMM)-normalized [26] and standardized heatmaps gene expression of: (**a**) Langer et al. (2019) [24] and (b) Shytaj et al. (2020) [25]. Both (**a**) and (**b**) display the 8 down-regulated genes and the first 20 up-regulated genes ranked by percentage of false prediction (pfp)-adjusted *p*-value. (**c**) Stripchart of average log2(Fold-change) of genes in infected cells when compared to uninfected/mock-infected cells identified by rank product method. Red dots in (**c**) represent differentially expressed genes with percentage of false prediction (pfp)-adjusted *p*-value < 0.05. The horizontal blue lines demarcate the |log2(Fold-change)| thresholds.

Among the 28,395 annotated genes, 28,165 genes were deemed to be not differentially expressed; among the remaining 230 genes, 22 did not pass the |log2(fold-change)| > 1 condition. Therefore, the remaining 208 genes were deemed as maDEGs during HIV-1 infection. Two hundred were up-regulated in infected cells when compared to uninfected/mock-infected cells and eight were down-regulated in infected cells when compared to uninfected/mock-infected cells. Figure 1 contains heatmaps of trimmed mean of M-values (TMM)-normalized [26] and standardized gene expression among the studies samples as well as stripcharts displaying the distribution of gene expression among the samples and pfp-adjusted *p*-values and log2(Fold-change) across genes.

The comparison of our 208 maDEGs with other predicted genes found in three studies [15,16,17,18] and two databases [19,20,21,22] revealed low concordance (Table 2). The one-sided binomial tests revealed that almost all intersections were within the rates expected by random chance. However, interestingly, the maDEGs list included 64 genes already known to interact with HIV-1 according to the HIV-1 Human Interaction Database [19,20,21]. The complete list of maDEGs is displayed in Table S2 alongside average log2(fold-changes) and comparison with previous studies and public databases.

The GO analysis revealed enrichment of 923 pathways involved in immune response, cell proliferation, cell adhesion, apoptosis, inflammation. Noticeably, Wnt, Notch and MAPK/ERK pathway signaling also were present. A summary of the GO analysis is displayed in Table 3. The complete list is displayed in Table S3.

## 4. Discussion

Immunological mechanisms play a crucial role in the HIV-1 infection process and the immunogenetic aspects in its modulation remain to be investigated [27]. In this context, we observed differential expression of genes at various points of the viral pathogenic process, such as activation of anti- and pro-viral mechanisms and induction of changes in cell structure.

In the initial stages of viral invasion of lymphoid tissues, the innate immune system is responsible for inducing the first antiviral response. Pathogen-associated molecular patterns receptors (PRRs) [24,25,28], such as *TLR7*, which were up-regulated in our analyses (GO:0045087 “innate immune response”), are responsible for identifying the pathogen initially [29]. They induce signaling pathways that regulate immune system-related genes, such as *TRIM* gene family [30,31]. This activation leads to transcription of important pro-inflammatory cytokines mRNA, such as IFN-γ [32], TNF-α [33] and other related genes such as *IFNB1* (GO:0045321 “leukocyte activation”) and *TNFSF4* (GO:0006954 “inflammatory response”), some of which were also up-regulated according to our meta-analysis. The induced pro-inflammatory milieu attracts other immune cell populations to the infection site. CCL7, CCL2, CCL3 and others are overexpressed during HIV-1 infection, contributing to the secretion of more pro-inflammatory cytokines, leading to chronicity of the infectious process observed in HIV-positive individuals [34,35,36].

Moreover, our GO analysis confirmed the role of some important pathways involved in HIV-1 infection: ERK/MAPK and Wnt. The ERK/MAPK pathway (extracellular signal-regulated kinases/mitogen-activated protein kinases) is an important regulator of IL-2, IL-10, and TNF-α cytokines expression [37]. Indeed, there is evidence showing that inhibition of this pathway suppresses viral replication and cytopathic effects in lymphocytes, probably via reverse transcription and viral integration impairment [38]. The Wnt/β-catenin pathway represses both basal and Tat-mediated HIV-1 transcription [39], perhaps by sequestering Tat in the cytoplasm [40,41]. We observed the up-regulation of *FZD4*, the gene of a frizzled class receptor as well as *WNT11* (GO:0016055 “Wnt signaling pathway”). Notch and Wnt pathways are interconnected in some cellular processes, with Notch being associated with T cell survival and quiescence [42]. Therefore, the activation of these pathways in the early infection may signify the establishment of an HIV-1 reservoir.

The expression of attachment proteins that facilitate migration through the endothelium and vasodilation are necessary for these cells to reach the infection site, as evidenced by, for example, *APOD, SDC2, CXCK11* and *VCAM1* in our analyses (GO:0050900 “leukocyte migration”). During cell migration, the interaction with adhesion molecules causes several changes in cell structure, so these cells can reach tissues [43,44]. Moreover, initial and final stages of retroviral cycle require changes in cell morphology [45,46], justifying the up-regulated genes observed in maintenance regulatory proteins in the cytoskeleton, such as *MAP1B*, *WNT11* and others (GO:0007010 “cytoskeleton organization”).

The establishment of infection also leads to other immunogenetic regulation process. An important aspect of this process concerns the immunological synapses that occur between neighboring cells. We identified the upregulation of Notch pathway-related genes *ROBO1, DLL4, DLL1, GATA2, JAG1* and *MDK* (GO:0007219 “Notch signaling pathway”), which act as co-activators in the stimulation of the NF-κB pathway, which induces IL-2 production, responsible for proliferation and activation of CD4+ T lymphocytes and is associated with latency suppression mechanisms and initiation of viral transcription [42,47]. The NF-κB (nuclear factor kappa B) pathway is exploited by HIV-1 to activate its transcription, by having NF-κB binding sites in its long terminal repeats (LTRs) [48].

We observed that 64 genes among our maDEGs already had annotated roles in HIV-1, since they were curated in the specialized HIV-1 Human Interaction Database [19,20,21] (Table S2). All of them participated in various biological process pathways (roughly the same as displayed in Table 3) as revealed by our GO analysis. Additionally, we detected three long non-coding RNA curated by RNAcentral [22] among our maDEGs: *WAKMAR2*, *MIR3945HG* and *WDR86-AS1*. Apparently, none of them have been associated with HIV-1 pathogenesis yet, although some MIR non-coding RNA genes have been involved in HIV-1 latency establishment [49,50].

We recognize some limitations in our study, which are similar to the ones evidenced in our previous review [51]: first, the data come from studies using cell lines or infected cells, which could bias gene expression profiles. Second, the process of HIV-1 infection establishment is a highly stochastic phenomenon. It is possible that the virus only infects a fraction of the cells which could also introduce bias into RNA expression profiles [52,53]. Besides that, HIV-1 transcriptional initiation tends to be lower in unstimulated CD4+ T cells or PBMCs and higher in activated CD4+ T cells [54]. Third, there were differences in the study design such as the clonality of virus used to experimentally infect cells (genetic variants in the virus genome may affect its capacity to activate viral transcription [55]) and differences of timepoints of RNA extraction and sequencing, for example. Fourth, and we share this limitation with every study of gene expression, modulation of transcription does not perfectly correlate with protein abundance, due to the cellular post-transcriptional and translational/post-translational regulatory processes [53]. Therefore, we tried to not become too speculative about the results. Thus, we selected pathways to present in the discussion that already have recognized roles in HIV replication and life cycle, to be as close to biological significance as possible. We believe that our cross-referencing helped gauge the relative contribution of some important pathways for HIV-1 early pathogenesis.

## 5. Conclusions

The methodology of RNA-Seq contributed to the discovery of mechanisms of cellular processes. In our study, we performed a reappraisal of transcriptomic libraries, identified a subset of genes consistently upregulated during HIV-1 infection and reviewed major genes and pathways involved in key steps of the pathogenic as well as immune response processes. Our approach allowed us to detect three long non-coding RNA, namely *WAKMAR2*, *MIR3945HG* and *WDR86-AS*, never associated before with HIV-1 pathogenesis. Therefore, we believe that our analyses contribute to provide insights, confirmatory and novel, useful for better understanding of HIV-1 infection dynamics.

## Figures and Tables

**Table 1:** Summary of studies included in the meta-analysis of gene expression profiles of CD4+ T infected with HIV-1 in vitro.

Study	PRJ, GSE IDs	Selected Samples	Control:Infected Ratio
Langer et al., 2019 [24]	PRJNA482835, GSE117655	16	1:3
Shytaj et al., 2020 [25]	PRJNA524856, GSE127468	6	1:1

**Table 2:** Cross-reference of individual studies with the meta-analysis results and other studies involving HIV-1 replication and life cycle, alongside HIV Human Interaction Database and RNAcentral, a database of non-coding RNA. The binomial distribution was used to test the null hypothesis that the intersection of the original source with the meta-analysis list is equal or less than expected by chance via one-sided tests.

Reference or Database	Unique Genes	Observed Intersection	Expected Intersection	*p*-Value
Konig et al., 2008 [16] ^a^	406	4	3	0.35
Zhou et al., 2008 [17]	264	2	2	0.60
Yeung et al., 2009 [18]	262	3	2	0.32
HIV interaction database [19,20,21] ^b^	4667	64	34	<0.001
RNAcentral [22] ^b^	7972	3	58	1.00

^a^ includes some genes detected by the Brass et al. 2008 study [15]. ^b^ Data retrieved as of 13 August 2020.

**Table 3:** Selected enriched pathways reported by gene ontology (GO) analysis of differentially expressed genes identified through meta-analysis of gene expression profiles of CD4+ T cells infected with HIV-1 in vivo.

Pathways and Ranks	GO ID	Term	FDR-Adjusted *p*-Value
Immune response			
1	GO:0006958	complement activation, classical pathway	6.4 × 10^−46^
9	GO:0002449	lymphocyte mediated immunity	1.2 × 10^−41^
12	GO:0006955	immune response	7.8 × 10^−39^
Cell proliferation			
97	GO:0008283	cell population proliferation	2.9 × 10^−14^
116	GO:0042127	regulation of cell population proliferation	5.0 × 10^−12^
351	GO:0046651	lymphocyte proliferation	0.00004
Cell adhesion			
151	GO:0007155	cell adhesion	5.9 × 10^−9^
826	GO:0007159	leukocyte cell–cell adhesion	0.0136
Cell migration			
23	GO:0016477	cell migration	1.2 × 10^−31^
39	GO:0050900	leukocyte migration	2.1 × 10^−28^
Apoptosis			
216	GO:0012501	programmed cell death	1.1 × 10^−6^
225	GO:0008219	cell death	1.7 × 10^−6^
234	GO:0042981	regulation of apoptotic process	2.5 × 10^−6^
Inflammation			
259	GO:0050727	regulation of inflammatory response	5.1 × 10^−6^
611	GO:0002526	acute inflammatory response	0.001
Wnt signaling			
441	GO:0016055	Wnt signaling pathway	0.0002
444	GO:0198738	cell–cell signaling by wnt	0.0002
580	GO:0060070	canonical Wnt signaling pathway	0.0010
Notch signaling			
591	GO:0008593	regulation of Notch signaling pathway	0.001
688	GO:0007219	Notch signaling pathway	0.003
ERK/MAPK signaling			
648	GO:0000165	MAPK cascade	0.002
691	GO:0043406	positive regulation of MAP kinase activity	0.003
886	GO:0070371	ERK1 and ERK2 cascade	0.031

## Data Availability

Data sharing not applicable.

## Supplementary Materials

Supplementary Materials can be found at https://www.mdpi.com/1999-4915/13/2/244/s1, Table S1: Detailed sample list of the studies included in the meta-analysis of gene expression profiles of CD4+ T cells infected with HIV-1 in vitro, Table S2: Results of the meta-analysis of differential gene expression analysis. Average, per-gene log2(fold-change) values (sorted by pooled *p*-value) are displayed alongside cross-referencing with other studies involving HIV replication and life cycle, alongside HIV Human Interaction Database and RNAcentral, a database of non-coding RNA., Table S3: Gene ontology (GO) analysis results sorted by FDR-adjusted *p*-value. The 211 DEGs identified by the meta-analysis were used as the input, Script S1: code for simulation of gene lists and binomial test for intersection between gene lists.
